# Pleomorphic Adenoma of the Parotid Gland and Ipsilateral Thyroid Incidentaloma: Report of A Rare Case With Review of Literature

**Published:** 2020-05-31

**Authors:** F Candelori, A Minni, A Greco, A Scarpa, C Cassandro, M Cavaliere, M Bisognox, E Cassandro, M de Vincentiis, M Ralli

**Affiliations:** 1 Department of Sense Organs, University Sapienza of Rome, Italy; 2 Department of Medicine and Surgery, University of Salerno, Salerno, Italy; 3 Department of Surgical Sciences, University of Turin, Turin, Italy; 4 Department of Otorhinolaryngology, University Hospital “San Giovanni di Dio e Ruggi D‘Aragona”, Salerno, Italy; 5 Department of Oral and Maxillo-Facial Surgery, University Sapienza of Rome, Italy

**Keywords:** Pleomorphic adenoma, Thyroid incidentaloma, Benign salivary gland tumors, Parotidectomy, Benign salivary gland tumors, multinodular goiter

## Abstract

**Background:**

Pleomorphic adenomas are benign tumors of the salivary glands that mainly affect the lower pole of the superficial lobe of the parotid gland. The term “pleomorphic” refers to the epithelial and connective origin of the mass. The clinical presentation is typically that of asymptomatic swelling which increases in volume. Therapy consists in surgical removal of the tumor mass by parotidectomy with nerve preservation.

**Case details:**

This clinical case describes an interesting case of pleomorphic adenoma of the parotid gland in a 62-year-old patient. The patient presented with a long history of an asymptomatic mildly worsening swelling of the left parotid region. The peculiarity of the clinical case is the dimension of the adenoma (9x5x9 cm) and the presence of a thyroid incidentaloma (TI), consisting of a thyroid multinodular goiter composed of nodules, the largest of which measured 8 cm in diameter. This mass dislocated the laryngotracheal axis, compressed the larynx and caused the reduction of the respiratory space, making orotracheal intubation difficult and determining the need to perform a tracheotomy.

**Conclusion:**

Benign pleomorphic adenomas can potentially reach large sizes if untreated. Socio-economic problems may be the reason for late diagnosis.

## I. INTRODUCTION

Salivary gland tumors represent 1–3% of head and neck cancer, they mainly affect the parotid gland and 70–80% are adenomas (1). Among benign neoplasms, the most frequent histotype is pleomorphic adenoma (PA). PA is more common in women with a male:female ratio of 1:2 and is more prevalent between the fourth and sixth decade of life (2–6).

PA is a mixed tumor that originates from epidermoid and myoepithelial cells; it includes mesenchymal tissues (myxoid, chondroid, mucoid) and epithelial tissues usually with adenomatous appearance (ducts, tubules, berries), hence the term “mixed tumor” (7, 8). PA is characterized by a slow but progressive growth and, if untreated, can reach a significant size (9), similarly to other benign head and neck masses such as lipoma (10). The diagnosis of PA is based on both clinical and radiological exams, including imaging techniques such as B-mode Ultrasound, Magnetic Resonance Imaging (MRI) and Computed Tomography (CT), followed by fine needle aspiration (FNA) for preoperatory cytological diagnosis (11, 12).

PA can undergo malignant transformation to carcinoma ex-pleomorphic adenoma (13–15); rarely it metastasizes without malignant transformation (16).

Treatment of PA is surgical; recurrence may occur mainly due to intraoperative multifunctional cellular effusions and cellular dissemination caused by difficulty in completing removal of the pseudocapsule (17). Historically, the incidence of recurrence was estimated between 20% and 40%; today, with the most modern parotidectomy techniques, recurrence is estimated around 2.5% (18–20). The management of recurrent pleomorphic adenoma of the parotid gland is challenging because the tumor is often multinodular and can be associated with lesions to the facial nerve (21, 22).

## II. Case Report

A 62-year-old female patient was admitted to the Otolaryngology Department of our university hospital reporting a painless swelling of the parotid region appeared several years earlier and slowly increased in volume.

The patient was suffering from high blood pressure, severe obesity (BMI: 37), permanent atrial fibrillation and iatrogenic hypothyroidism. The patient regularly assumed nebivolol, furosemide and levothyroxine. In 1999, the patient had undergone a left hemithyroidectomy for thyroid nodularity.

Physical examination showed facial symmetry with normal movements of the face and eyes. The swelling in the left parotid region had a multilobular appearance and an elastic consistency; it was not painful on palpation and the overlying skin were hyperemic.

**Figure 1:**
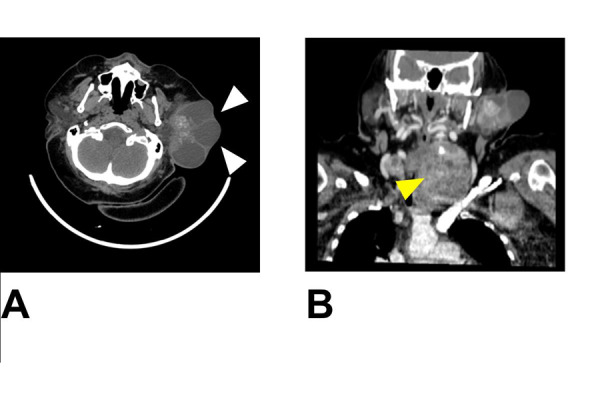
Facial Computed tomography scan with contrast agent of the head and neck. A) solid hypervascularized mass in the superficial lobe of the left parotid gland measuring 9x5x9 cm (white arrows. B) a solid hypervascularized mass in the left thyroid lobe with multiple focal calcifications over 8 cm in diameter (yellow arrow).

Fibrolaryngoscopy showed a paralysis of the left hemilarynx, probably due to the previous thyroid surgery; respiratory space was reduced. The patient underwent CT scan with contrast agent that detected a solid hypervascularized mass in the superficial lobe of the left parotid gland measuring 9x5x9 cm (Figure 1 A). CT scan also revealed a thyroid incidentaloma (TI), a solid hypervascularized mass in the left thyroid lobe with multiple focal calcifications over 8 cm in diameter consistent with multinodular goiter (MG) (Figure 1 B).

The trachea was strongly deflected on the right side, in association with compression of the left pyriform sinus and ipsilateral hemilarynx. Pathological lymph nodes were found in the left submandibular region. Ultrasound-guided FNA confirmed the presence of a multinodular goiter with a TIR 2 lesion (non-malignant / benign lesion) according to the 2014 Italian Consensus for Classification and Reporting of Thyroid Cytology classification (22).

**Figure 2:**
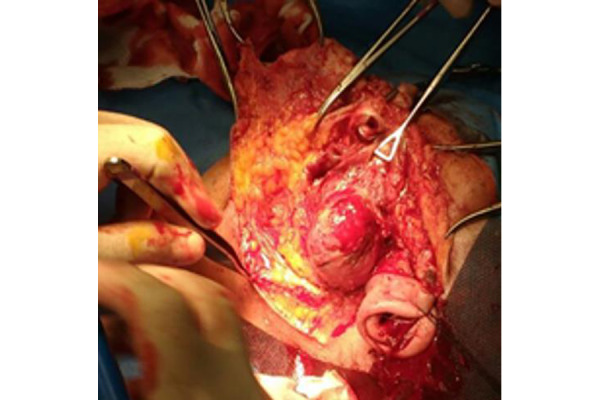
Intraoperative image showing a capsulated, solid and cystic mass in the superficial lobe of the left parotid gland.

Patient underwent a combined parotidectomy and thyroidectomy (Figure 2); a temporary tracheotomy was performed due to the preexisting conditions of the patient.

Histological examination of the parotid specimen revealed a capsulated, solid and cystic mass characterized by the presence of a wide squamous component containing partially calcified corneal cysts and by multifocal proliferation of epithelial cells, which formed structures that resembled glandular lumina and spindle-shaped myoepithelial cells bearing pale cytoplasm. A hyaline and chondromyxoid extracellular matrix was present. There was evidence of multiple granulomatous inflammation foci containing multinucleated giant cells, even within the capsule. The features of chondromyxoid foci and glando-duttal differentiation and the low growth rate (ki67 <5%) confirmed the diagnosis of PA with extensive squamous metaplasia. The histological exam also revealed reactive nodes, negative for tumoral invasion. Histological examination of the thyroid mass showed multiple colloid nodules in both lobes, characteristic of multinodular goiter (23–25). The parenchyma had histological features of macro and microfollicular stroma, with areas of focal necrosis sclerosis and inflammatory infiltration.

The patient had no postoperative complications; tracheostomy was removed 2 weeks after surgery and showed no recurrence of disease at regular follow up visits (1, 3, 6, 12 and 18 months after surgery).

## III. DISCUSSION

This case report shows an obese patient with a large PA who presented in a late stage of the disease. In addition, the patient presented a contextual finding of a TI, a MG whose largest nodule reached 8 cm in diameter. The presence of vocal fold paralysis probably due to previous hemithyroidectomy, the very voluminous MG and the displacement of the laryngotracheal axis complicated the surgical intervention.

PA or benign mixed tumor is the most common salivary gland neoplasm; in 80% of cases it involves the parotid gland, in 8% the submandibular gland, and in 6% the minor salivary glands (5). Generally, PA involves the lower pole of the superficial lobe of the parotid gland, is not painful and presents solid consistency (5, 21, 26). Facial nerve palsy is a rare occurrence (27), and was not found in this patient despite the large size of the parotid mass.

Although encapsulated, in some locations the capsule is not fully developed and expansile growth produces protrusions into the surrounding gland, which may lead to recurrences if the tumor is enucleated. In the present case, a radical parotidectomy was performed with removal of the pseudocapsule; no recurrence of the parotid mass was found during follow up.

The dominant histologic feature found in this case of PA is the great heterogeneity. The epithelial elements resembling ductal cells or myoepithelial cells are arranged in duct formations, acini, irregular tubules, strands, or sheets of cells. These elements are typically dispersed within a mesenchyme-like background of loose myxoid tissue containing islands of cartilage and, rarely, foci of bone. Sometimes, the epithelial cells form well-developed ducts lined by cuboidal to columnar cells with an underlying layer of deeply chromatic, small myoepithelial cells. In other cases, there may be strands or sheets of myoepithelial cells. Islands of well-differentiated squamous epithelium may also be present. In most cases there is no epithelial dysplasia or evident mitotic activity. There is no difference in biologic behavior between the tumors composed largely of epithelial elements and those composed largely of seemingly mesenchymal elements (28).

Malignant transformation occurs in nearly 3% of cases, is usually linked a long-standing, untreated condition and has to be suspected in case of pain, ulceration, spontaneous bleeding, superficial and/or deep tissue invasion, rapid growth and facial nerve alterations (29, 30).

In conclusion, the present case shows a peculiar situation in which a PA and TI were found in the same patient at a very late stage. PA may reach significant sizes if untreated, and socio-economic problems can be a reason for late diagnosis and treatment.

## Abbreviations

PA: pleomorphic adenoma
TI: thyroid incidentaloma
CT: computerized tomography
MRI: magnetic resonance imaging
MG: multinodular goiter
FNA: fine needle aspiration
